# Cirrhotic Human Liver Extracellular Matrix 3D Scaffolds Promote Smad-Dependent TGF-β1 Epithelial Mesenchymal Transition

**DOI:** 10.3390/cells9010083

**Published:** 2019-12-28

**Authors:** Giuseppe Mazza, Andrea Telese, Walid Al-Akkad, Luca Frenguelli, Ana Levi, Martina Marrali, Lisa Longato, Kessarin Thanapirom, Maria Giovanna Vilia, Benedetta Lombardi, Claire Crowley, Mark Crawford, Morten A. Karsdal, Diana J. Leeming, Giusi Marrone, Katrin Bottcher, Benjamin Robinson, Armando Del Rio Hernandez, Domenico Tamburrino, Gabriele Spoletini, Massimo Malago, Andrew R. Hall, Jasminka Godovac-Zimmermann, Tu Vinh Luong, Paolo De Coppi, Massimo Pinzani, Krista Rombouts

**Affiliations:** 1 Regenerative Medicine & Fibrosis Group, Institute for Liver and Digestive Health, University College London (UCL), London NW3 2PF, UK; a.telese@ucl.ac.uk (A.T.); walid.al-akkad@ucl.ac.uk (W.A.-A.); l.frenguelli@ucl.ac.uk (L.F.); ani.levi@outlook.com (A.L.); martina.marrali.16@alumni.ucl.ac.uk (M.M.); k.thanapirom@ucl.ac.uk (K.T.); m.vilia@ucl.ac.uk (M.G.V.); giusimarrone@gmail.com (G.M.); katrin.boettcher17@gmail.com (K.B.); tamburrino.domenico@hsr.it (D.T.); gabriele.spoletini@gmail.com (G.S.); massimo.malago@nhs.net (M.M.); andrewhall1@nhs.net (A.R.H.); tuvinh.luong@nhs.net (T.V.L.); m.pinzani@ucl.ac.uk (M.P.); 2 Engitix Ltd., London NW3 2PF, UK; lisa.longato@engitix.com; 3 Proteomics and Molecular Cell Dynamics, Centre for Nephrology, School of Life and Medical Sciences, University College London, London NW3 2PF, UK; benedettalombardi@hotmail.com (B.L.); m.crawford@ucl.ac.uk (M.C.); j.godovac-zimmermann@ucl.ac.uk (J.G.-Z.); 4 Stem Cells and Regenerative Medicine Section, Developmental Biology and Cancer Programme, UCL Institute for Child Health, Great Ormond Street Hospital, University College London, London WC1N 3JH, UK; claire.crowley.09@ucl.ac.uk (C.C.); paolo.decoppi@gosh.nhs.uk (P.D.C.); 5 Nordic Bioscience, Biomarkers & Research, Herlev Hovedgade 205-207, 2730 Herlev, Denmark; mk@nordicbio.com (M.A.K.); djl@nordicbio.com (D.J.L.); 6 Department of Bioengineering, Cellular and Molecular Biomechanics, Imperial College, London SW7 2AZ, UK; benjaminkennethrobinson@gmail.com (B.R.); a.del-rio-hernandez@imperial.ac.uk (A.D.R.H.); 7 Sheila Sherlock Liver Centre, Royal Free London NHS Foundation Trust, London NW3 2PF, UK; 8 Specialist Neonatal and Paediatric Surgery at Great Ormond Street Hospital, London WC1N 3JH, UK

**Keywords:** hepatocellular carcinoma (HCC), tumor microenvironment (TME), 3-dimensional (3D) platform, 3D ECM scaffolds, decellularized human liver, decellularized extracellular matrix (dECM), proteomics, hepatocellular carcinoma cells, Transforming growth factor beta1 (TGF-β1), tissue engineering

## Abstract

An altered liver microenvironment characterized by a dysregulated extracellular matrix (ECM) supports the development and progression of hepatocellular carcinoma (HCC). The development of experimental platforms able to reproduce these physio-pathological conditions is essential in order to identify and validate new therapeutic targets for HCC. The aim of this work was to validate a new in vitro model based on engineering three-dimensional (3D) healthy and cirrhotic human liver scaffolds with HCC cells recreating the micro-environmental features favoring HCC. Healthy and cirrhotic human livers ECM scaffolds were developed using a high shear stress oscillation-decellularization procedure. The scaffolds bio-physical/bio-chemical properties were analyzed by qualitative and quantitative approaches. Cirrhotic 3D scaffolds were characterized by biomechanical properties and microarchitecture typical of the native cirrhotic tissue. Proteomic analysis was employed on decellularized 3D scaffolds and showed specific enriched proteins in cirrhotic ECM in comparison to healthy ECM proteins. Cell repopulation of cirrhotic scaffolds highlighted a unique up-regulation in genes related to epithelial to mesenchymal transition (EMT) and TGFβ signaling. This was also supported by the presence and release of higher concentration of endogenous TGFβ1 in cirrhotic scaffolds in comparison to healthy scaffolds. Fibronectin secretion was significantly upregulated in cells grown in cirrhotic scaffolds in comparison to cells engrafted in healthy scaffolds. TGFβ1 induced the phosphorylation of canonical proteins Smad2/3, which was ECM scaffold-dependent. Important, TGFβ1-induced phosphorylation of Smad2/3 was significantly reduced and ECM scaffold-independent when pre/simultaneously treated with the TGFβ-R1 kinase inhibitor Galunisertib. In conclusion, the inherent features of cirrhotic human liver ECM micro-environment were dissected and characterized for the first time as key pro-carcinogenic components in HCC development.

## 1. Introduction

ECM being the non-cellular component present within all tissues and organs provides not only essential physical scaffolding for the cellular constituents, but also initiates crucial biochemical and biomechanical signals. These signals are required for tissue morphogenesis, differentiation, and homeostasis. Indeed, the ECM is a highly dynamic structure that is constantly remodeled by cells that are constantly rebuilding and modifying the ECM through synthesis, degradation, and reassembly of its 3D structure. These processes are complex and need to be tightly regulated to maintain tissue homeostasis, especially in response to injury associated with chronic diseases, as a dysregulated ECM remodeling represents a key feature leading to disease progression [1].

The idea of mutual interaction between ECM and cells has been always identified as key factor in orchestrating physiological and pathological conditions. However, it remains largely undefined whether extracellular support is to be seen as a passive structure or as a bioactive micro-environment affecting the cross-talk. Early two-dimensional (2D) cell culture technologies could not reproduce this complex 3D microenvironment, thus showing certain limitations when applied to disease modelling and drug discovery [2]. Consequently, technologies developed in recent years attempt to reproduce the tissue microenvironment by using 3D structures for cell culture. However, most of these technologies employ inert component materials with clear limitations in fully recapitulating human physiology and physiopathology, especially when studying the complex interactions regulating drug response in human diseases [3,4].

Hepatocellular carcinoma (HCC) is a primary malignancy, which arises mostly as a consequence of pre-existing chronic liver diseases due to chronic viral hepatitis, excessive alcohol consumption, metabolic, and immune-mediated diseases [5]. HCC has become one of the leading causes of cancer deaths worldwide and, regardless of the etiology, occurs predominantly in patients with advanced chronic liver disease/cirrhosis [6,7]. Chronic hepatocellular necrosis, inflammation, oxidative stress, and dysregulated extracellular matrix (ECM) deposition are responsible for genetic alterations and deregulation of signaling pathways [8,9,10], all favoring the development of HCC. Besides liver transplantation, resection, and loco-regional ablation, the pharmacological treatment of HCC is characterized by few options, which are reserved to the more advanced phase of HCC with still very limited success [11,12].

The discovery of pharmacological targets for the treatment of HCC has been traditionally carried out by employing in vitro experiments on HCC cell lines and animal models [13]. 

The aim of this work was to overcome the current methodological limitations by establishing a new in vitro experimental platform based on engineering 3D normal or cirrhotic human liver ECM scaffolds with HCC cells in order to re-create the biochemical and biomechanical tissue microenvironment favoring the development and the progression of HCC. Thus, a key end-point of this platform optimization and evaluation was to investigate the cellular and molecular pathways of TGFβ1, a multifunctional cytokine provided with a dual pro-neoplastic action by promoting directly HCC development and progression, or indirectly by inducing a pro-inflammatory and pro-fibrogenic microenvironment and facilitating HCC immune escape [14]. In addition, TGFβ1 has been found to exert a tumor promoter function, which coincides with the progression of the tumor and the presence of EMT and metastasis [14]. Although the role of TGFβ1 has been extensively investigated with a focus on Smad (canonical) [14] and non-Smad (non-canonical) [15] signaling pathways, the impact of human ECM proteins, matricellular components, and their topology on the working mechanisms of TGFβ1 are still poorly defined. 

Our findings demonstrate that healthy and cirrhotic ECM retained architectural, biochemical, and biomechanical features able to affect cell behavior and function with distinct patterns. In particular, these two platforms differed in terms of unique ECM components with specific bio-active cues and microarchitecture, which can dictate specific gene expression and signaling pathways leading to the activation of the EMT program as well as the response towards TGFβ1. 

## 2. Materials and Methods

Detailed description of materials and methods can be found in the Supplementary Materials.

### 2.1. Decellularization Protocol for Healthy and Cirrhotic Human 3D Liver Scaffolds

Healthy and cirrhotic liver was obtained under local ethics from the UCL Royal Free BioBank Ethical Review Committee (NRES Rec Reference: 11/WA/0077). Informed consent was given by each patient taking part in the study [16,17]. Native liver tissue (5mm × 5 mm × 5mm) was thawed in a water bath at 37 °C for 45 min followed by addition of 1mL of 1× phosphate buffered saline (PBS) and incubated for another 15 min at 37 °C. Tissues were transferred in decellularization solution and placed in QIAGEN TissueLyser II and frequency of oscillation was set at 30 Hz. Tissue decellularization was achieved by several cycles of agitation in decellularization solutions such as deionized water (Milli-Q^®^ ultrapure water, VWR, Leicestershire, UK) reagent mixture (detergents and enzyme), and PBS. The same concentrations were used for both healthy liver (unsuitable for liver transplantation) and cirrhotic liver tissue (explant primary sclerosing cholangitis), however the protocols were optimized according to the tissue and cycles were repeated according to the saturations of detergents (solution turns darker due to the debris) (Supplementary Materials Table S1). Immunohistochemistry was performed against Collagen type I, collagen type III, collagen type IV, fibronectin, and laminin (Supplementary Materials and Table S2).

### 2.2. Scanning Electron Microscopy (SEM)

Samples were fixed in 2.5% glutaraldehyde. Next, samples were washed 3 times with 1% PBS, placed in a 0.5% PBS solution containing 25% sucrose and 10% glycerol for 2 h, and then snap frozen in liquid nitrogen and processed for SEM as previously described [16,17] (Supplementary Materials).

### 2.3. Second Harmonic Generation (SHG) and Imaging

Briefly, both native tissue and decellularized liver scaffolds were cryoprotected and sections were then set in a mold and covered in OCT cold embedding media and frozen as previously described [17] (Supplementary Materials).

### 2.4. Atomic Force Microscopy (AFM)

Sample preparation: Tissue samples for AFM measurement were prepared by taking tissue slices from a cube of liver tissue as previously described [16,17] (Supplementary Materials).

### 2.5. Collagen Proportionate Area Analysis

The collagen proportionate area (CPA) was measured as described previously [18]. Briefly, image capture was accomplished with a Canon Powershot A640 digital camera and digital image analysis (DIA) used a visual basic script for Zeiss Axiovision (version 4.8.2., Carl Zeiss Ltd., Cambridge, UK). The algorithm uses a binary segmentation of RGB color channels to distinguish liver tissue from collagen and an editing step was included. This allows manual editing of confounding artefacts such as major blood vessels and liver capsule. The collagen proportionate area was calculated as the area occupied by the collagen as a proportion of the area of the whole parenchyma and expressed as a percentage [18].

### 2.6. Proteomic Analysis

Protein extraction, separation, and in-gel protein digestion: Proteins were extracted from healthy and cirrhotic liver tissues before and after the decellularization process, for every 3 biological repeats processed in triplicate. Mass spectrometry and protein analysis was performed as previously described [19] (Supplementary Materials).

### 2.7. Bioengineering

Cell culture: Hep G2 ATCC^®^ HB-8065 were cultured using Gibco™ Minimum Essential Media supplemented with 10% fetal bovine serum, Penicillin-Streptomycin Gibco™ 1:100, 0.1 mM/L non-essential amino acids, 1.0 mM/L sodium pyruvate i.e., complete culture medium. Sterilization of liver scaffolds was performed as previously described [17]. 

Recellularization: The sterilized liver scaffolds were placed in a 48-well plate and incubated overnight in 1.4 mL of complete culture medium in a humidified incubator at 37 °C, 5% CO_2_ (day -1). Scaffolds were repopulated with cells (2 × 10^6^ in 20 µL) using the drop-on technique [17]. The cultures were placed in a humidified incubator at 37 °C and 5% CO_2_. After 3 h of incubation, 1.4 mL of complete media was added to each well (day 0). Cell culture media was changed and collected every 3 days for further analysis. 

Treatment: HepG2 cells grown on both types of 3D scaffolds were treated on day 7 with: (1) TGFβ1 (5–10 ng/mL, R&D SYSTEMS); (2) simultaneously treated with TGFβ1 and TGFβ-R1 kinase inhibitor Galunisertib (10 µM); or (3) pretreated with TGFβ1 for 6 h followed by TGFβ-R1 kinase inhibitor Galunisertib (10 µM) (LY2157299 Selleck Chemical) up to 48 h. Next, cell culture medium was collected and stored at −80 °C, whereas 1 scaffold of each condition was prepared for histological analysis and 4 scaffolds were snap-frozen and stored at −80 °C for further analysis.

### 2.8. Gene Expression

EMT RT2 Profiler PCR Array: Total RNA was extracted using TRIzol reagent and RNeasy Universal Mini Kit (Qiagen, Manchester, UK) according to the manufacturer’s guidelines and Supplementary Materials.

### 2.9. Protein Synthesis and Secretion

Intracellular protein analysis: The repopulated snap-frozen scaffolds were washed in PBS1X and cells were lysed by placing scaffolds in radio-immunoprecipitation assay buffer (RIPA Buffer, Sigma Aldrich, Dorset, UK) [20] with 5 mm diameter stainless-steel bead magnetic beads (Qiagen). The tubes were agitated for 5 min at full speed (50 cycles per second) using the TissueLyser (Qiagen). Total proteins were measured via micro-bicinchoninic (BCA) assay (Pierce, Rockford, IL, USA) and stored at −80 °C for further analysis [20]. A 7-plex signaling assay for cell lysates was obtained by combining the TGF-β Signaling 6-plex Magnetic bead kit (48-614MAG), with a total β-tubulin Magnetic bead MAPmate (46-713MAG) for normalization according to the manufacturer’s guidelines and detailed described in Supplementary Materials. 

Acellular scaffold protein extraction: Scaffolds were washed in PBS1X and lysed as described above. Samples were incubated on ice for 15 min and then centrifuged at 4 °C for 15 min at 14,000 rpm. The supernatant containing the protein lysate was recovered and total proteins were measured via micro-bicinchoninic (BCA) assay (Pierce, Rockford, IL, USA), Western blot analysis was performed with anti-TGF beta 1 antibody (ab92486, Abcam), and quantitative densitometric analysis was performed as previously described [21]. 

TGFβ1 release in acellular scaffolds. Extracellular TGFβ1 levels in the culture media were measured with Human TGFβ1 ELISA Kit (ab100647) (Abcam, Cambridge, UK) according to the manufacturer’s guidelines and Supplementary Materials. 

Fibronectin secretion: Secreted fibronectin levels were measured using an enzyme linked immunosorbent assay (ELISA), known as FBN-C, towards human fibronectin according to the manufacturer’s guidelines (Nordic Bioscience A/S, Herlev, Denmark) [22] (Supplementary Materials).

### 2.10. Statistical Analysis

For the significance of differentially expressed protein, the *p*-value Student’s *t*-test was used and the sig-B [23]. CPA data were considered non-parametric, distributions were described using median and quartiles, and differences in populations tested with the Mann–Whitney U test. Unpaired Student T test was used for the EMT profiler array dataset and genes that presented expression levels with a fold change of 2 and *p* ≤ 0.05 were considered to be differentially expressed.

## 3. Results

### 3.1. Cirrhotic Liver Tissue Scaffold Characterization

The decellularization of the cirrhotic tissue was obtained by adapting the protocol described previously for the decellularization of the 3D healthy human liver scaffolds [17] (Supplementary Materials Table S1). 

**Figure 1:**
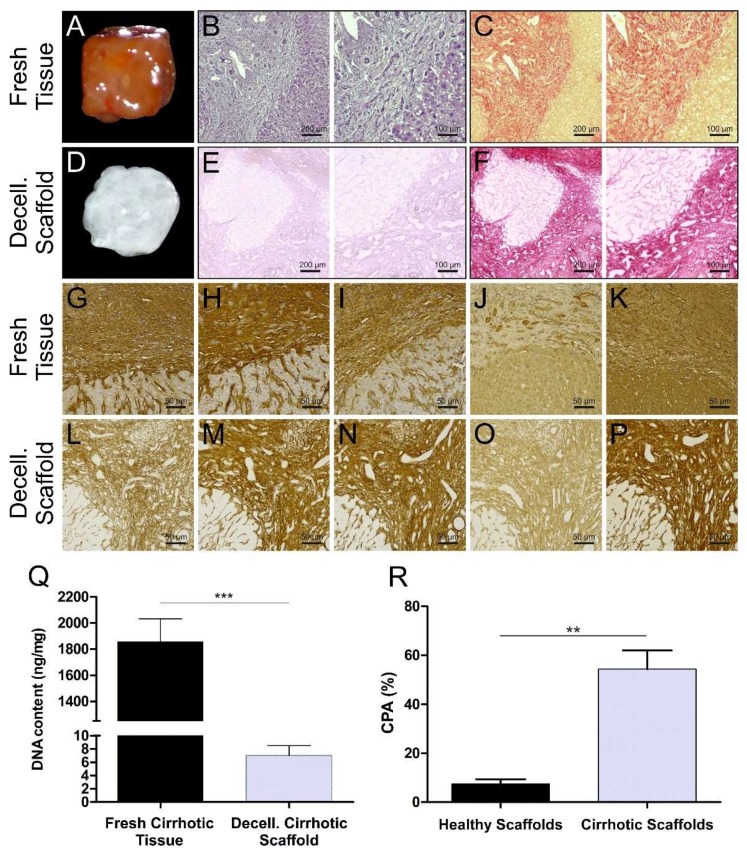
Macroscopic characterization of decellularization of human liver 3D scaffolds. (**A**) Macroscopic appearance of native cirrhotic liver 3D scaffold before and (**D**) after decellularization. (**B**,**C**) Histological comparison of cirrhotic native tissue and (**E**,**F**) decellularized 3D scaffold after staining with Haematoxylin and Eosin (H&E) showing acellularity (E) and Sirius Red (SR) collagen preservation (F), respectively (scale bars, 100–200 μm). (**G**–**P**) Distribution of several ECM proteins; collagen I, collagen III, collagen IV, fibronectin, and laminin, respectively, evaluated by immunohistochemistry showing consistency between the native tissue (top panel, **G**–**K**) and decellularized 3D cirrhotic scaffolds (bottom panel, **L**–P) (scale bars, 50 μm). (**Q**) DNA quantification showing significant elimination of DNA in the native fresh tissue versus 3D cirrhotic scaffolds (*n* = 4 for each condition, *** *p* < 0.0005 native tissue versus 3D scaffold). (**R**) Collagen proportional area (CPA) showed a significant difference between healthy and cirrhotic 3D scaffolds (** *p* < 0.021: Median normal 7.5%, LQ-UQ 3.8%–11.1% versus cirrhotic median 53.7%, LQ-UQ 40.6%–69%).

The resultant cirrhotic scaffolds were characterized by translucent appearance when compared to native tissues (Figure 1A compared to 1D). As part of quality control, the absence of residual cellular components in the ECM scaffold was confirmed by Haematoxylin and Eosin staining (Figure 1B compared to 1E). The histological evaluation by Sirius Red (SR) staining showed that the general liver tissue architecture of the cirrhotic liver was preserved with the typical nodular architecture and fibrous septa (Figure 1C compared to 1F), and different compared to the previously described healthy liver 3D architecture [17]. Immunohistochemistry staining showed the presence and the distribution pattern of the major key ECM components after the decellularization process. Collagen type I, collagen type III, collagen type IV, fibronectin, and laminin were all maintained in the acellular tissue (Figure 1L–P, bottom panel) when compared to the native liver tissue (Figure 1G–K, upper panel). Moreover, the DNA content was below the accepted threshold of 50 ng/mg of tissue [24] with the average amount of DNA of 7 ± 3 ng/mg (SD = ±3; *n* = 4) after decellularization i.e., significantly and sufficiently lower compared to the native tissue (Figure 1Q). Furthermore, the quantitative measurement of collagen content was performed by determination of Collagen Proportion Area (CPA) in order to quantify fibrillar collagens. CPA showed a significant difference between healthy and cirrhotic 3D scaffolds (*p* < 0.021: Median normal 7.5%, LQ-UQ 3.8%–11.1% versus cirrhotic median 53.7%, LQ-UQ 40.6%–69%) (Figure 1R). 

**Figure 2:**
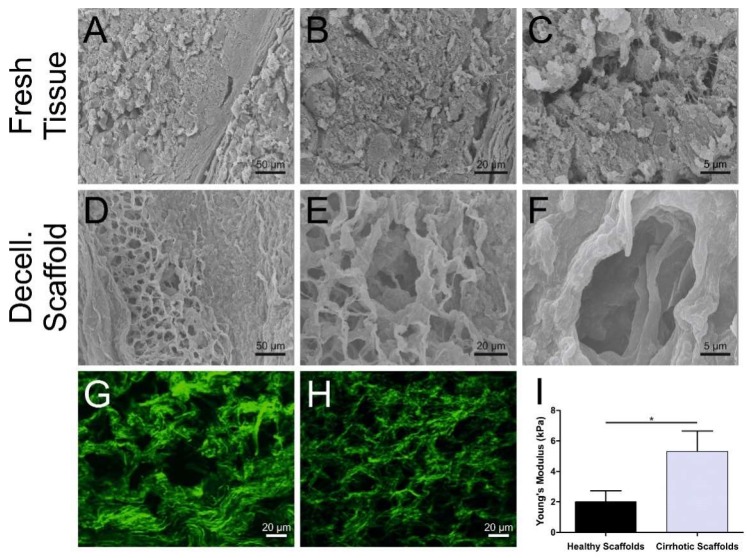
Microscopic characterization of decellularization of human liver 3D scaffolds. (**A**–**F**) SEM imaging of native tissue (top panel, **A**–**C**) and decellularized 3D cirrhotic scaffolds (bottom panel, **D**–**F**) showing the preservation of the nodular fibrotic ECM and conservation of the hepatocyte pockets (scale bars 50 µm, 10 µm, and 5 μm for each condition). (**G**) Second harmonic generation analysis of fibrillar collagens structure (green) of healthy decellularized 3D scaffolds with (**H**) more abundant and compact fibrillar collagen structures present in cirrhotic decellularized 3D scaffolds (scale bars, 20 μm). (**I**) AFM comparison of tissue stiffness between healthy decellularized 3D scaffolds and cirrhotic decellularized 3D scaffolds: Healthy scaffold 2.03 ± 0.59 kPa and cirrhotic scaffold 5.25 ± 1.09 kPa. Data are expressed as mean ± SEM, * *p* = 0.0118, unpaired *t*-test).

Next, scanning electron microscopy was used to evaluate the impact of the decellularization process on the 3D microstructure of the cirrhotic ECM (Figure 2A–F). The decellularization procedure did not affect the overall 3D architecture of cirrhotic tissues, in comparison to the fresh tissue (Figure 2A–C) as the resultant cirrhotic scaffolds were characterized by preserved cirrhotic-like nodules, increased network of ECM filaments, as well as a thick network of ECM proteins surrounding the hepatocyte pocket (Figure 2D–F) and this in comparison to healthy liver 3D scaffolds (Supplementary Figure S1). In addition, collagen fiber alignment and deposition were further evaluated by second harmonic generation (SHG), which confirmed an increased thickening of fibrillary collagens in the decellularized cirrhotic nodules (Figure 2G) when compared with healthy 3D scaffolds (Figure 2H). Next, the biomechanical characterization of human liver cirrhotic 3D scaffolds in comparison to healthy liver scaffolds was assessed by atomic force microscopy (AFM) in order to measure tissue stiffness. Calculation of Young’s modulus from the AFM force curves demonstrated a significant difference between the stiffness of decellularized healthy 3D scaffolds and decellularized cirrhotic 3D scaffolds (*p* = 0.0118) (Figure 2I). As previously described in other studies on biological scaffolds [25], the Young’s modulus calculated for the decellularized healthy liver tissue was 2.03 ± 0.59 kPa (mean ± SEM). In contrast, the Young’s modules calculated for the decellularized cirrhotic liver 3D scaffolds was 5.25 ± 1.09 kPa (mean ± SEM) i.e., approximately three times greater than that of healthy liver 3D scaffolds (Figure 2I). Overall, these data confirmed that the 3D liver microanatomy, ultrastructure, and biomechanical properties of both healthy and cirrhotic scaffolds were fully preserved after the decellularization procedure.

### 3.2. Proteomic Analysis of Healthy and Cirrhotic 3D Liver Scaffolds

**Figure 3:**
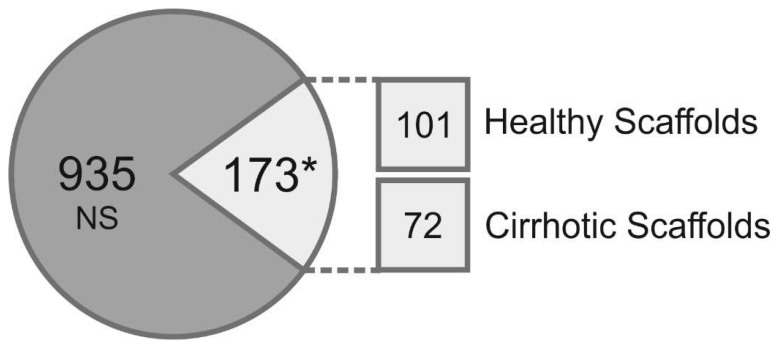
Proteomic analysis of healthy liver 3D scaffolds and cirrhotic liver 3D scaffolds. The composition of the ECM in decellularized healthy and cirrhotic liver scaffolds was qualitatively and quantitatively investigated by a label free proteomic analysis (*n* = 3, three biological repeats, processed in triplicate for each condition). A relative quantitative analysis was performed on 1108 proteins showing 173 proteins significantly changed (* *p* < 0.05) between healthy scaffold and cirrhotic 3D scaffold ECM. In 3D healthy scaffolds, 101 proteins were overexpressed, whereas 72 proteins were significantly changed in decellularized 3D cirrhotic liver scaffolds.

The composition of the ECM (“matrisome”) [26] in decellularized healthy and cirrhotic liver scaffolds was qualitatively and quantitatively investigated by a label-free proteomic analysis in three biological repeats processed in triplicate for each condition. By considering the proteins present in at least two replicates in each condition, a total of 1108 proteins were identified and were shared between both healthy and cirrhotic scaffolds. By performing a relative quantitative analysis (Max Quant), a total of 173 proteins significantly changed (Student’s *t*-test *p* < 0.05) (Figure 3) between the healthy scaffold and the cirrhotic scaffold matrisomes. 

In the healthy scaffold dataset, we identified 101 proteins, which were more abundantly present in comparison to the cirrhotic scaffold dataset (Table 1). 

In the decellularized cirrhotic scaffold dataset, 72 proteins were identified, which were quantitatively increased in comparison to the healthy scaffold (Table 2). ECM proteins such as COL5A1, COL10A1, and the crosslinking enzyme LOXL1; TGFβ1-related proteins such as TGFB1I1, LTBP1, and LTBP4; and integrin-related proteins FLNA, FBN1, and FBLN5 were significantly increased in cirrhotic 3D scaffolds. 

Overall, this analysis confirmed the preservation of key ECM components in the acellular healthy and cirrhotic scaffold and identified proteins specifically enriched in the cirrhotic ECM.

### 3.3. Cirrhotic ECM Drives EMT Induction

**Figure 4:**
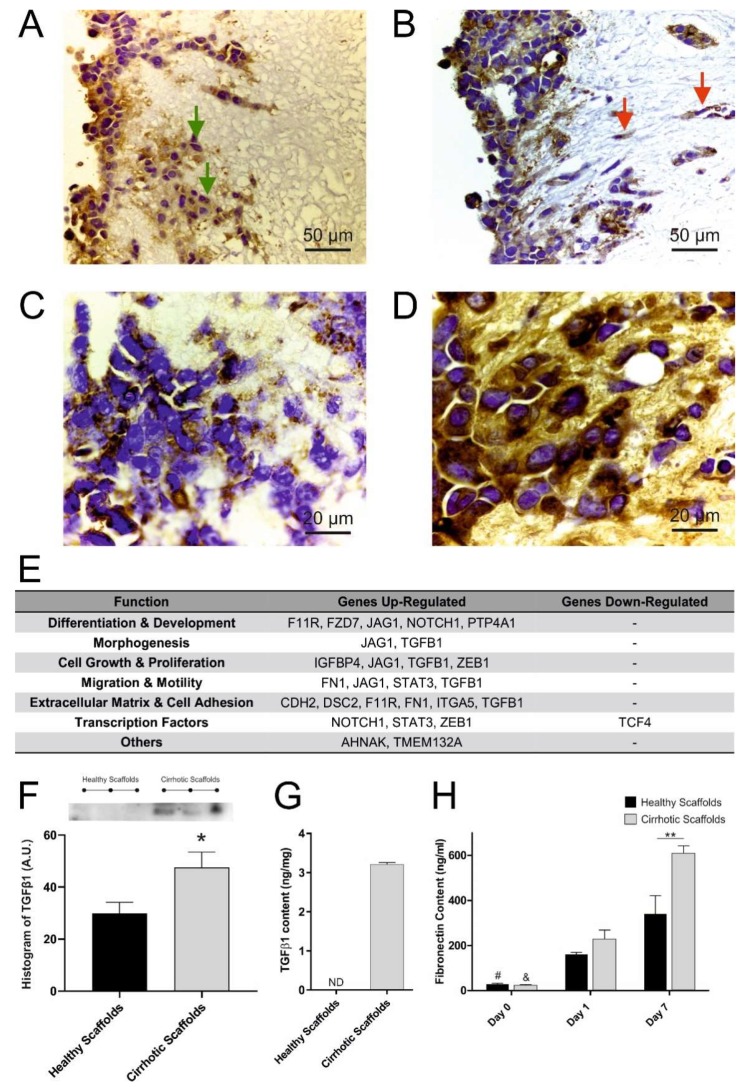
Cirrhotic 3D ECM scaffolds drive EMT in HepG2 cells. Epithelial cell adhesion molecule (EPCAM) immunohistochemistry of HepG2 cells engrafted in (**A**) healthy 3D scaffolds (green arrows) and (**B**) cirrhotic 3D scaffolds (scale bar 50 µm) (red arrows showing more mesenchymal phenotype), and alpha fetoprotein (AFP) immunohistochemistry of HepG2 cells engrafted in (**C**) healthy 3D scaffolds and (**D**) cirrhotic 3D scaffolds (scale bar 20 µm). (**E**) Overview of RT2 Profiler PCR Array for EMT performed in HepG2 cells grown on healthy and cirrhotic 3D scaffolds, which showed significant upregulation of EMT-related genes in HepG2 cells engrafted in 3D cirrhotic scaffolds (fold change of 2 and *p* ≤ 0.05). (**F**) Western blot analysis demonstrating significant increase in TGFβ1 protein expression in acellular cirrhotic 3D scaffolds in comparison to healthy 3D acellular scaffolds (*n* = 3 scaffolds per condition). (**G**) Overnight endogenous TGFβ1 release assessed in culture media from acellular 3D healthy scaffolds (0 ng/mL) and acellular 3D cirrhotic scaffolds (3.3 ng/mL) (*n* = 3 for each condition, pooled and measured in duplicate). (**H**) Fibronectin secretion was assessed by employing FBN-C ELISA and demonstrated a significant upregulation in FN1 secretion by HepG2 cells grown on cirrhotic scaffolds in comparison to HepG2 cells grown on healthy scaffolds at day 7 (** *p* < 0.005), and versus day 0 for healthy scaffolds (# *p* < 0.005), and versus day 0 for cirrhotic scaffolds for all time points measured (& *p* < 0.005). Data are expressed as mean +/-SD of the amount of FN from four scaffolds per condition (*n* = 4 scaffolds per condition).

In a next set of experiments, the role of disease-specific liver ECM scaffolds in modulating cell behavior was investigated. To explore the effect of different 3D human ECM substrates in modulating EMT, healthy and cirrhotic scaffolds were reseeded with HepG2 cells. Immunohistochemistry of epithelial cell adhesion molecule (EPCAM) expression showed that cells engrafting the cirrhotic 3D scaffolds were characterized by a mesenchymal-like phenotype and expressed more alpha fetoprotein (AFP) than cells grown in healthy 3D scaffolds (Figure 4D). Furthermore, the EMT RT2 PCR profiler demonstrated that cells reseeded on cirrhotic 3D scaffolds, significantly increased the expression of genes involved in differentiation, development, and morphogenesis such as JAG1 and Notch1; growth and proliferation such as TGFβ1 and ZEB1; ECM and cell adhesion with FN1 and CDH2; and motility and cancer EMT-driven aggressiveness with STAT3, FN1, and TGFβ1 (Figure 4E). These data, together with the previously obtained proteomics matrisome data of decellularized scaffolds (Figure 3), further indicated that the disease-specific ECM composition can modify gene expression and that cirrhotic ECM scaffolds favors EMT-driven pro-carcinogenic signaling pathways. 

Considering the presence of TGFβ1 associated proteins in the cirrhotic matrisome and TGFβ1 associated response genes, Western blot analysis was performed and demonstrated that TGFβ1 was significantly retained in cirrhotic ECM in comparison to healthy ECM scaffolds (Figure 4F). Furthermore, a TGFβ1 ELISA was employed in acellular scaffolds to further explore the possibility that the TGFβ1, embedded in the scaffold, could be released in the culture medium. Data demonstrate a significant release of TGFβ1 in the media of cirrhotic 3D scaffolds when compared to the healthy scaffolds (3.3 ng/mL versus 0 ng/mL, respectively) (Figure 4G). 

Fibronectin (FN1) is a major scavenger and delivery system for TGFβ1-LTBP1 complex [27], and the gene expression was upregulated in HepG2 cells during the ECM-induced EMT process (Figure 4E). Therefore, the protein expression and secretion was further explored in bioengineered constructs and along these lines, a significant increase in FN1 secretion was observed in cirrhotic scaffolds reseeded with HepG2 over a time period of seven days with a significant increase in bioengineered 3D cirrhotic scaffolds for each time point under investigation (Figure 4H). Overall, these data indicate that FN1, a neo-epitope ECM marker of fibrogenesis [22,28], was released differently when cells were grown on 3D healthy or 3D cirrhotic ECM scaffolds.

### 3.4. ECM Composition Affects TGFβ1-Induced Phosphorylation of Smad2/Smad3 Signaling Pathway

**Figure 5:**
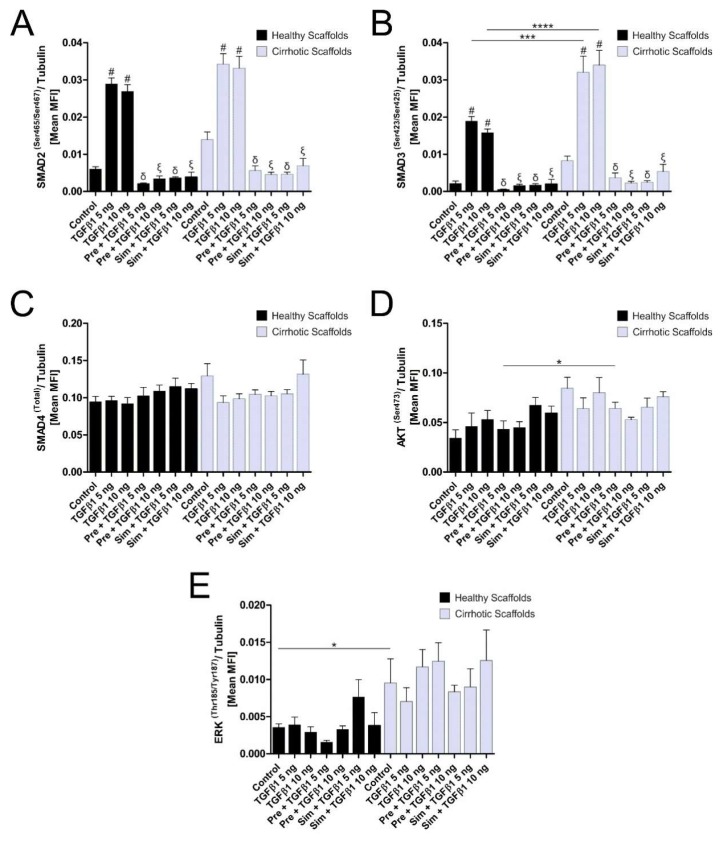
A specific ECM composition dictates TGFβ1-induced p-Smad2 and p-Smad3 signaling pathway in HepG2 cells grown in both types of 3D scaffolds. A 7-plex Luminex assay was used to quantify the relative levels of phosphorylated (**A**) SMAD2 (Ser465/Ser467), (**B**) SMAD3 (Ser423/Ser425), (**C**) total SMAD4, (**D**) AKT (Ser473), and (**E**) ERK (Thr185/Tyr187) normalized to tubulin protein levels (*n* = 4 samples per condition). Exogenous TGFβ1 treatment induced robust upregulation in P-Smad2 and P-Smad3 levels in both healthy and cirrhotic scaffolds compared with controls (# = *p* < 0.05 versus control). The levels of P-Smad3 in TGFβ1-treated samples were further increased in 3D cirrhotic scaffolds versus 3D healthy scaffolds (*** *p* = 0.005 for TGFβ1 5ng in cirrhotic scaffolds versus TGFβ1 5ng in healthy scaffolds, **** *p* < 0.0001 for TGFβ1 10ng in cirrhotic scaffold versus TGFβ1 10ng in healthy scaffold). The robust induction in SMAD2 and-3 phosphorylation induced by TGFβ1 treatment was prevented by either pre-treatment (Pre) or simultaneous treatment (Sim) with the TGFβ-R1 kinase inhibitor Galunisertib (10 µM) (δ = *p* < 0.05 versus TGFβ1 5ng/mL, ξ = *p* < 0.05 versus TGFβ1 10ng/mL). *= *p* < 0.05 for the other comparisons.

The evidence so far suggested that gene expression was affected by tissue- and disease-specific ECM components present in healthy and cirrhotic liver 3D scaffolds. In this set of experiments, TGFβ1 downstream signaling pathways were investigated in HepG2 cells grown in both types of 3D scaffolds, with and without TGFβ1 treatment (5 ng/mL and 10 ng/mL). Moreover, cells were pre-treated or simultaneous treated with Galunisertib, a TGFβ-R type 1 inhibitor used in phase 2 trials as second-line HCC treatment [29,30]. Phosphorylation of Smad2 and Smad3 increased significantly in both types of 3D scaffolds upon TGFβ1 treatment in comparison to non-treated conditions (Figure 5A,B). Furthermore, phosphorylation of Smad3 was significantly increased when cells were grown in 3D cirrhotic scaffolds upon treatment with TGFβ1 in comparison to cells grown in 3D healthy scaffolds (Figure 5B). Total Smad4 did not significantly change in all conditions under investigation (Figure 5C). Galunisertib showed to significantly inhibit the TGFβ1-induced phosphorylation of Smad2 and Smad3, indicating that the 3D liver scaffolds can be used as drug screening platform. We further explored the possible involvement of the non-canonical TGFβ1 signaling pathway effector proteins such as the phosphorylation of Akt and Erk. Phosphorylation of Akt showed an increased expression in non-treated HepG2 cells grown in 3D cirrhotic scaffolds versus 3D healthy scaffolds control cells (Figure 5D) without any further significant changes upon treatment with TGFβ1 and/or Galunisertib. Phosphorylation of Erk was significantly increased in 3D cirrhotic scaffolds pre-incubated with Galunisertib followed by TGFβ1 exposure, when compared to the same treatment in 3D healthy scaffolds (Figure 5E). Overall, these data reinforce the concept that disease-ECM specificity plays a key role in favoring the activation/phosphorylation of the canonical proteins Smad2 and Smad3 in TGFβ1-induced signaling pathway.

## 4. Discussion

The results of the work presented herein demonstrate that the ECM of human cirrhotic liver is characterized by unique bioactive features promoting the progression of HCC. Up to now, the possibility of dissecting the role of the pre-carcinogenic acellular tumor microenvironment has been hampered by the lack of adequate models. Indeed, the lack of insights into disease-specific matrisome components, ECM biochemical, and biomechanical properties has challenged our understanding of how cells interact with, and respond to, the microenvironment for decades [31,32,33].

In order to overcome these methodological issues, we adapted our previous protocol established for the development of small-scale healthy human liver 3D scaffolds [17] to obtain cirrhotic human scaffolds. The resultant cirrhotic liver 3D scaffold was characterized by the preservation of the essential biochemical, biomechanical, physical, and topographical properties typical of native human cirrhotic liver tissue including thick ECM fibers surrounding and deforming the hepatocyte-free space and perisinusoidal structures in addition to porto-central fibrotic septa. These changes were further associated with differences in ECM scaffold stiffness and fibrillary collagen amount/distribution typical of the cirrhotic liver [9]. The matrisome of the decellularized healthy and cirrhotic liver 3D scaffolds was further investigated by a label-free proteomic analysis identifying disease-specific ECM components. In particular, this analysis revealed a unique enrichment in ECM proteins associated to the TGFβ/ECM related pathways such as Hic5, LTBP-1, and-4, FBN1, Fibulin-1 and 2, and Fibrillin-1 in cirrhotic scaffolds. Therefore, we postulated that the cirrhotic liver ECM is branded by a bioactive environment potentially favoring HCC development and progression.

To this end, we engineered both cirrhotic and healthy liver ECM scaffolds with the HepG2, a hepatoblastoma cell line known to reproduce many of the features of polarized human hepatocytes [34], which was primarily employed to explore the pro-carcinogenicity of cirrhotic liver ECM. Cirrhotic liver 3D scaffolds induced a strong increase in EMT when compared with bioengineered 3D scaffolds obtained from healthy liver. These findings confirmed our overall hypothesis that pathological changes in the hepatic ECM microarchitecture, biochemical, and mechanical structure can favor the development of more aggressive neoplastic features. 

With the previous observations that the cirrhotic ECM contains an abundant signature of TGFβ- and ECM-related proteins and it favors a pro-carcinogenic microenvironment, we next addressed the possibility that TGFβ1 was endogenously retained in larger amounts in cirrhotic ECM scaffolds. Indeed, acellular cirrhotic 3D scaffolds contained and released TGFβ1 into the medium and this was not detected when analyzing the acellular healthy ECM. Future studies will define, in detail, the mechanisms by which TGFβ1 is released from the scaffolds, including the detection and characterization of active proteases and or protease inhibitors. We also investigated the regulation of FN1, a key ECM component responsible for binding and stably releasing TGFβ1 in the tissue microenvironment [27], of which release showed to be upregulated in cirrhotic liver scaffolds. This observation further reinforces the concept that the cirrhotic ECM is provided not only with a remarkable bioavailability of TGFβ1, but also with the necessary machinery for its activation in the tissue microenvironment [35]. 

Further, the key role of TGFβ in modulating cell biology in both ECM tissues was also confirmed by the evaluation of the TGFβ downstream signaling pathways [14,15] in engineered liver ECM scaffolds. Notably, the phosphorylation patterns of TGFβ1 signaling proteins Smad 2 and specifically Smad3 were remarkably different between cells reseeded into healthy and cirrhotic liver scaffolds and upon TGFβ1 treatment. Furthermore, the applicability of the 3D platform was investigated by treating the 3D bioengineered scaffolds with Galunisertib, a TGFβ-R type 1 inhibitor used in phase 2 trials as second-line HCC treatment [29,30]. The treatment significantly inhibits the TGFβ1-induced phosphorylation of Smad2 and Smad3, thus indicating that the 3D liver scaffolds in vitro model can be used as drug screening platform.

Overall, the data provided by the present work highlight that the unique disease-specific ECM environment affects both cell differentiation and function i.e., recapitulating those processes that are important during progressive fibrogenesis, HCC development, and progression [36,37,38]. 

In conclusion, we herein describe, for the first time to our knowledge, a novel 3D human liver ECM platform derived from human cirrhotic liver. The in vitro biocompatibility and bioactivity of disease-specific ECM was tested by bioengineering with cancer cells, for its potential in recapitulating leading pathways in HCC development and progression. The inherent features of cirrhotic ECM were for the first time dissected and characterized as key pro-carcinogenic components in HCC. In addition, the role of TGFβ1, a key player in driving EMT progress during cancer development, was further characterized in terms of its relationship with the cirrhotic ECM. This new methodology will likely expand the knowledge of the role of tissue-specific and disease-specific extracellular components in fibrosis and cancer cell progression with the possibility of identifying new potential therapeutic targets for drug development.

## Figures and Tables

**Table 1:** Significantly abundant proteins in decellularized healthy scaffolds.

Gene Name	*p*-Value a = 10^−5^, b = 10^−4^, c = 10^−3^, d = 10^−2^	Gene Name	*p*-Value a = 10^−5^, b = 10^−4^, c = 10^−3^, d = 10^−2^	Gene Name	*p*-Value a = 10^−5^, b = 10^−4^, c = 10^−3^, d = 10^−2^
FGG	4.9 × 10 ^a^	NUDC	1.4 × 10 ^d^	THNSL1	3.0 × 10 ^d^
FKBP4	6.1 × 10 ^a^	HSP90AA1	1.6 × 10 ^d^	SEPHS2	3.0 × 10 ^d^
COL6A6	1.4 × 10 ^b^	CSNK1A1	1.7 × 10 ^d^	BDH1	3.0 × 10 ^d^
NECAP2	5.9 × 10 ^b^	RBM39	1.7 × 10 ^d^	COL12A1	3.1 × 10 ^d^
ECM1	7.2 × 10 ^b^	SEC24C	1.7 × 10 ^d^	PSMD2	3.1 × 10 ^d^
SCYL2	9.1 × 10 ^b^	MMAB	1.7 × 10 ^d^	PAPSS2	3.1 × 10 ^d^
FGB	9.1 × 10 ^b^	FKBP5	1.7 × 10 ^d^	MARS	3.2 × 10 ^d^
CCT4	9.9 × 10 ^b^	ARAF	1.7 × 10 ^d^	SEC31A	3.2 × 10 ^d^
GSPT1	1.1 × 10 ^c^	GNE	1.8 × 10 ^d^	LGMN	3.3 × 10 ^d^
HUWE1	1.1 × 10 ^c^	CCT3	1.8 × 10 ^d^	HSPA4L	3.3 × 10 ^d^
HSPA4	1.2 × 10 ^c^	SAR1A	1.8 × 10 ^d^	EPRS	3.3 × 10 ^d^
SEC24B	1.6 × 10 ^c^	SDSL	1.9 × 10 ^d^	CHORDC1	3.4 × 10 ^d^
HSPD1	1.8 × 10 ^c^	LUM	1.9 × 10 ^d^	FGL1	3.6 × 10 ^d^
LONP1	1.9 × 10 ^c^	NADK2	2.0 × 10 ^d^	COL6A1	3.6 × 10 ^d^
PSMD11	2.8 × 10 ^c^	COASY	2.0 × 10 ^d^	TRAP1	3.6 × 10 ^d^
BGN	3.5 × 10 ^c^	EIF4G2	2.0 × 10 ^d^	HNRNPK	3.7 × 10 ^d^
TCP1	3.5 × 10 ^c^	FN1	2.0 × 10 ^d^	CBS	3.8 × 10 ^d^
UBR4	3.7 × 10 ^c^	ACAD11	2.1 × 10 ^d^	COPG1	3.8 × 10 ^d^
CCT2	4.3 × 10 ^c^	APPL1	2.1 × 10 ^d^	RECQL	3.9 × 10 ^d^
AASS	4.4 × 10 ^c^	EIF2B5	2.2 × 10 ^d^	MON2	3.9 × 10 ^d^
RAB2A	4.6 × 10 ^c^	GALT	2.3 × 10 ^d^	COL6A2	3.9 × 10 ^d^
CCT7	5.0 × 10 ^c^	EIF4G1	2.3 × 10 ^d^	TOLLIP	3.9 × 10 ^d^
POLDIP2	5.3 × 10 ^c^	TPD52L1	2.4 × 10 ^d^	NAGS	4.0 × 10 ^d^
EEF1G	5.9 × 10 ^c^	SERPINF2	2.4 × 10 ^d^	CHST13	4.3 × 10 ^d^
EIF3D	7.9 × 10 ^c^	RRAGC	2.4 × 10 ^d^	TAGLN2	4.5 × 10 ^d^
FGA	1.0 × 10 ^d^	PCYT2	2.5 × 10 ^d^	SEC23B	4.6 × 10 ^d^
CYB5R1	1.0 × 10 ^d^	GYS2	2.5 × 10 ^d^	SRP72	4.6 × 10 ^d^
PRB1	1.1 × 10 ^d^	AHSA1	2.5 × 10 ^d^	IDH3A	4.7 × 10 ^d^
EIF2S3	1.1 × 10 ^d^	CTSZ	2.6 × 10 ^d^	HSP90AB1	4.7 × 10 ^d^
MLYCD	1.2 × 10 ^d^	DARS	2.6 × 10 ^d^	PRMT1	4.8 × 10 ^d^
USP9X	1.2 × 10 ^d^	DDX21	2.7 × 10 ^d^	SPRYD4	4.8 × 10 ^d^
TFG	1.3 × 10 ^d^	NAMPT	2.9 × 10 ^d^	ACAD9	4.8 × 10 ^d^
TUBB2B	1.4 × 10 ^d^	NEDD4	2.9 × 10 ^d^	DCXR	4.8 × 10 ^d^
BZW1	1.4 × 10 ^d^	FTL	3.0 × 10 ^d^		

**Table 2:** Significantly abundant proteins in decellularized cirrhotic scaffolds.

Gene Name	*p*-Valuea a = 10^−5^, b = 10^−4^, c = 10^−3^, d = 10^−2^	Gene Name	*p*-Valuea a = 10^−5^, b = 10^−4^, c = 10^−3^, d = 10^−2^	Gene Name	*p*-Valuea a = 10^−5^, b = 10^−4^, c = 10^−3^, d = 10^−2^
IGKV3-11	2.1 × 10 ^a^	MYO1C	1.2 × 10 ^d^	SDHA	3.2 × 10 ^d^
BST1	1.3 × 10 ^b^	GPX3	1.3 × 10 ^d^	IGKV1D-33	3.3 × 10 ^d^
IGKV2D-28	3.5 × 10 ^b^	CTSS	1.4 × 10 ^d^	FLNA	3.4 × 10 ^d^
NDUFB3	6.2 × 10 ^b^	SEC22B	1.7 × 10 ^d^	LAMA2	3.4 × 10 ^d^
DNAJB9	8.2 × 10 ^b^	MYOF	1.7 × 10 ^d^	CD47	3.4 × 10 ^d^
FHL2	1.4 × 10 ^c^	ADH1B	1.8 × 10 ^d^	EHD2	3.4 × 10 ^d^
TMEM43	1.4 × 10 ^c^	FBLN1	1.9 × 10 ^d^	CHST4	3.5 × 10 ^d^
EFEMP1	1.6 × 10 ^c^	TBL2	1.9 × 10 ^d^	EMILIN1	3.6 × 10 ^d^
LXN	1.6 × 10 ^c^	H3F3B	2.0 × 10 ^d^	NCKAP1	3.7 × 10 ^d^
IGKV1-5	1.7 × 10 ^c^	RRAS	2.0 × 10 ^d^	NOP56	3.7 × 10 ^d^
HSD17B12	2.6 × 10 ^c^	VCAN	2.0 × 10 ^d^	COL5A1	3.7 × 10 ^d^
CLTC	4.3 × 10 ^c^	HNRNPM	2.3 × 10 ^d^	ADH1A	3.7 × 10 ^d^
C1QB	4.9 × 10 ^c^	IGKV3-20	2.4 × 10 ^d^	IGLC3	3.9 × 10 ^d^
IGHG1	5.1 × 10 ^c^	TGFB1I1	2.4 × 10 ^d^	TNS3	4.0 × 10 ^d^
CTSG	5.5 × 10 ^c^	SERPINA1	2.5 × 10 ^d^	GNB1	4.0 × 10 ^d^
MFAP4	6.7 × 10 ^c^	GNAI2	2.6 × 10 ^d^	PKD1L1	4.1 × 10 ^d^
IGKV3-15	8.4 × 10 ^c^	VDAC2	2.7 × 10 ^d^	CAV1	4.4 × 10 ^d^
FBLN5	8.7 × 10 ^c^	FBN1	2.8 × 10 ^d^	MEMO1	4.4 × 10 ^d^
IGFBP7	9.2 × 10 ^c^	LTBP1	2.8 × 10 ^d^	HBB	4.4 × 10 ^d^
TRAM1	1.0 × 10 ^d^	APOC3	2.8 × 10 ^d^	LGALS3	4.5 × 10 ^d^
HLA-DRA	1.0 × 10 ^d^	FLOT1	2.9 × 10 ^d^	LTBP4	4.6 × 10 ^d^
S100A9	1.0 × 10 ^d^	THSD4	3.1 × 10 ^d^	RPL10	4.7 × 10 ^d^
FLOT2	1.0 × 10 ^d^	PDLIM7	3.2 × 10 ^d^	AKR7L	4.8 × 10 ^d^
LOXL1	1.1 × 10 ^d^	FBLN2	3.2 × 10 ^d^	COL10A1	4.9 × 10 ^d^

## Supplementary Materials

The following are available online at https://www.mdpi.com/2073-4409/9/1/83/s1, Figure S1: Scanning Electron Microscopy of a decellularized 3D healthy scaffold. Table S1: Decellularization protocols followed for healthy and cirrhotic liver scaffolds. Table S2: Antibodies used when performing immunohistochemistry and western blot analysis.

## References

[1] Ng M.R., Brugge J.S. (2009). A stiff blow from the stroma: Collagen crosslinking drives tumor progression. Cancer Cell.

[2] Bhadriraju K., Chen C.S. (2002). Engineering cellular microenvironments to improve cell-based drug testing. Drug Discov. Today.

[3] Freires I.A., Sardi J.C., de Castro R.D., Rosalen P.L. (2016). Alternative Animal and Non-Animal Models for Drug Discovery and Development: Bonus or Burden?. Pharm. Res..

[4] Shanks N., Greek R. (2008). Experimental use of nonhuman primates is not a simple problem. Nat. Med..

[5] Ferlay J., Soerjomataram I., Dikshit R., Eser S., Mathers C., Rebelo M., Parkin D.M., Forman D., Bray F. (2015). Cancer incidence and mortality worldwide: Sources, methods and major patterns in GLOBOCAN 2012. Int. J. Cancer.

[6] Bruix J., Gores G.J., Mazzaferro V. (2014). Hepatocellular carcinoma: Clinical frontiers and perspectives. Gut.

[7] Llovet J.M., Zucman-Rossi J., Pikarsky E., Sangro B., Schwartz M., Sherman M., Gores G. (2016). Hepatocellular carcinoma. Nat. Rev. Dis. Primers.

[8] Hernandez-Gea V., Toffanin S., Friedman S.L., Llovet J.M. (2013). Role of the Microenvironment in the Pathogenesis and Treatment of Hepatocellular Carcinoma. Gastroenterology.

[9] Carloni V., Luong T.V., Rombouts K. (2014). Hepatic stellate cells and extracellular matrix in hepatocellular carcinoma: More complicated than ever. Liver Int..

[10] Giannelli G., Rani B., Dituri F., Cao Y., Palasciano G. (2014). Moving towards personalised therapy in patients with hepatocellular carcinoma: The role of the microenvironment. Gut.

[11] Nault J.C., Galle P.R., Marquardt J.U. (2018). The role of molecular enrichment on future therapies in hepatocellular carcinoma. J. Hepatol..

[12] Llovet J.M., Ricci S., Mazzaferro V., Hilgard P., Gane E., Blanc J.F., de Oliveira A.C., Santoro A., Raoul J.L., Forner A. (2008). Sorafenib in advanced hepatocellular carcinoma. N. Engl. J. Med..

[13] Zhang H.E., Henderson J.M., Gorrell M.D. (2019). Animal models for hepatocellular carcinoma. Biochim. Biophys. Acta. Mol. Basis Dis..

[14] Budi E.H., Duan D., Derynck R. (2017). Transforming Growth Factor-beta Receptors and Smads: Regulatory Complexity and Functional Versatility. Trends Cell Biol..

[15] Zhang Y.E. (2009). Non-Smad pathways in TGF-beta signaling. Cell Res..

[16] Mazza G., Rombouts K., Rennie Hall A., Urbani L., Vinh Luong T., Al-Akkad W., Longato L., Brown D., Maghsoudlou P., Dhillon A.P. (2015). Decellularized human liver as a natural 3D-scaffold for liver bioengineering and transplantation. Sci. Rep..

[17] Mazza G., Al-Akkad W., Telese A., Longato L., Urbani L., Robinson B., Hall A., Kong K., Frenguelli L., Marrone G. (2017). Rapid production of human liver scaffolds for functional tissue engineering by high shear stress oscillation-decellularization. Sci. Rep..

[18] Calvaruso V., Burroughs A.K., Standish R., Manousou P., Grillo F., Leandro G., Maimone S., Pleguezuelo M., Xirouchakis I., Guerrini G.P. (2009). Computer-assisted image analysis of liver collagen: Relationship to Ishak scoring and hepatic venous pressure gradient. Hepatology.

[19] Li Q., Uygun B.E., Geerts S., Ozer S., Scalf M., Gilpin S.E., Ott H.C., Yarmush M.L., Smith L.M., Welham N.V. (2016). Proteomic analysis of naturally-sourced biological scaffolds. Biomaterials.

[20] Longato L., Andreola F., Davies S.S., Roberts J.L., Fusai G., Pinzani M., Moore K., Rombouts K. (2017). Reactive gamma-ketoaldehydes as novel activators of hepatic stellate cells in vitro. Free Radic. Biol. Med..

[21] Marrone G., De Chiara F., Bottcher K., Levi A., Dhar D., Longato L., Mazza G., Zhang Z., Marrali M., Iglesias A.F. (2018). The AMPK-v-ATPase-pH axis: A key regulator of the pro-fibrogenic phenotype of human hepatic stellate cells. Hepatology.

[22] Bager C.L., Gudmann N., Willumsen N., Leeming D.J., Karsdal M.A., Bay-Jensen A.C., Hogdall E., Balslev I., He Y. (2016). Quantification of fibronectin as a method to assess ex vivo extracellular matrix remodeling. Biochem. Biophys. Res. Commun..

[23] Cox J., Mann M. (2008). MaxQuant enables high peptide identification rates, individualized p.p.b.-range mass accuracies and proteome-wide protein quantification. Nat. Biotechnol..

[24] Crapo P.M., Gilbert T.W., Badylak S.F. (2011). An overview of tissue and whole organ decellularization processes. Biomaterials.

[25] Goh S.K., Bertera S., Olsen P., Candiello J.E., Halfter W., Uechi G., Balasubramani M., Johnson S.A., Sicari B.M., Kollar E. (2013). Perfusion-decellularized pancreas as a natural 3D scaffold for pancreatic tissue and whole organ engineering. Biomaterials.

[26] Hynes R.O., Naba A. (2012). Overview of the matrisome--an inventory of extracellular matrix constituents and functions. Cold Spring Harb Perspect. Biol..

[27] Griggs L.A., Hassan N.T., Malik R.S., Griffin B.P., Martinez B.A., Elmore L.W., Lemmon C.A. (2017). Fibronectin fibrils regulate TGF-beta1-induced Epithelial-Mesenchymal Transition. Matrix Biol..

[28] Kim H., Park J., Kim Y., Sohn A., Yeo I., Yu S.J., Yoon J.H., Park T., Kim Y. (2017). Serum fibronectin distinguishes the early stages of hepatocellular carcinoma. Sci. Rep..

[29] Faivre S., Santoro A., Kelley R.K., Gane E., Costentin C.E., Gueorguieva I., Smith C., Cleverly A., Lahn M.M., Raymond E. (2019). Novel transforming growth factor beta receptor I kinase inhibitor galunisertib (LY2157299) in advanced hepatocellular carcinoma. Liver Int..

[30] Kelley R.K., Gane E., Assenat E., Siebler J., Galle P.R., Merle P., Hourmand I.O., Cleverly A., Zhao Y., Gueorguieva I. (2019). A Phase 2 Study of Galunisertib (TGF-beta1 Receptor Type I Inhibitor) and Sorafenib in Patients with Advanced Hepatocellular Carcinoma. Clin. Transl. Gastroenterol..

[31] Howlett A.R., Bissell M.J. (1993). The influence of tissue microenvironment (stroma and extracellular matrix) on the development and function of mammary epithelium. Epithelial Cell Biol..

[32] Bhat R., Bissell M.J. (2014). Of plasticity and specificity: Dialectics of the micro- and macro-environment and the organ phenotype. Wiley Interdiscip. Rev. Membr. Transp. Signal.

[33] Karsdal M.A., Manon-Jensen T., Genovese F., Kristensen J.H., Nielsen M.J., Sand J.M., Hansen N.U., Bay-Jensen A.C., Bager C.L., Krag A. (2015). Novel insights into the function and dynamics of extracellular matrix in liver fibrosis. Am. J. Physiol..

[34] Gissen P., Arias I.M. (2015). Structural and functional hepatocyte polarity and liver disease. J. Hepatol..

[35] Dituri F., Mancarella S., Cigliano A., Chieti A., Giannelli G. (2019). TGF-β as Multifaceted Orchestrator in HCC Progression: Signaling, EMT, Immune Microenvironment, and Novel Therapeutic Perspectives. Semin. Liver Dis..

[36] Zeisberg M., Kramer K., Sindhi N., Sarkar P., Upton M., Kalluri R. (2006). De-differentiation of primary human hepatocytes depends on the composition of specialized liver basement membrane. Mol. Cell. Biochem..

[37] Levental K.R., Yu H., Kass L., Lakins J.N., Egeblad M., Erler J.T., Fong S.F., Csiszar K., Giaccia A., Weninger W. (2009). Matrix crosslinking forces tumor progression by enhancing integrin signaling. Cell.

[38] Zhang D.Y., Friedman S.L. (2012). Fibrosis-dependent mechanisms of hepatocarcinogenesis. Hepatology.

