# Supplementary material for: Cirrhotic Human Liver Extracellular Matrix 3D Scaffolds Promote Smad-Dependent TGF-β1 Epithelial Mesenchymal Transition
Source: Cells. 2019 Dec 28;9(1):83. doi: 10.3390/cells9010083 (PMC7017194; doi:10.3390/cells9010083)
Supplement: Supplementary file 1 [file cells-09-00083-s001.zip › Giuseppe Mazza et al Suppl info materials and methods.pdf]

SUPPLEMENTARY INFORMATION: MATERIAL AND METHODS

Cirrhotic human liver ECM scaffolds promote carcinogenesis through an epithelial mesenchymal transition by Smad dependent-TGF-β1 pathway

Giuseppe Mazza[1*], Andrea Telese[1], Walid Al-Akkad[1], Luca Frenguelli[1], Ana Levi[1], Martina Marrali[1], Lisa Longato[1], Kessarin Thanapirom[1], Maria Giovanna Vilia[1], Benedetta Lombardi[2], Claire Crowley[5], Mark Crawford[2], Morten A Karsdal[7], Diana J Leeming[7], Giusi Marrone[1], Katrin Bottcher[1], Benjamin Robinson[3], Armando Del Rio Hernandez[3], Domenico Tamburrino[1], Gabriele Spoletini[1], Massimo Malago[1], Andrew R. Hall[1,4], Jasminka Godovac-Zimmermann[2], Tu Vinh Luong[1], Paolo De Coppi[5,6], Massimo Pinzani[1,4], Krista Rombouts[1*]

Sources of reagents. Unless otherwise specified, reagents were purchased from Sigma Aldrich, whereas immunohistochemistry reagents were purchased from Novocastra.

Tissue characterization.

Histochemistry and immunohistochemistry: Decellularized 3D scaffolds were fixed in 10% formalin at room temperature for at least 24 hours, processed and embedded in paraffin blocks as previously described [2]. The samples were cut (3 µm), dewaxed and rehydrated using xylene, IDA and water. The histochemical stainings were obtained by using Haematoxylin and Eosin (H&E) (Leica, Germany) and Picro-Sirius Red (SR) (Hopkin & Williams) (BDH Chemicals Ltd, Cellpath Ltd). Immunohistochemistry for collagen types I, III, IV, fibronectin, laminin and Ki67 was performed as previously described [1, 2]. Briefly, tissue was incubated in 0.5% Trypsin (MP Biomedical)/ 0.5% Chymotrypsin / 1% Calcium Chloride (BDH) in Tris buffered saline pH 7.6 (TBS) for 30 minutes at 37 °C. The slides were soaked in TBS with 0.04% Tween-20 for 5 minutes, blocked in peroxidase blocking solution for 5 minutes, washed in TBS for 5 minutes and then incubated for 1 hour in the primary antibodies (Supplementary Table 2). Slides were placed for 25 minutes in NovolinkTM post primary, 25 minutes in NovolinkTM polymer solution and developed with NovolinkTM 3,3′ di-amino-benzidine with a 5 minutes wash in TBS with 0.04% Tween-20 between each step. The stained slides were dehydrated and mounted using DPX mounting medium (Leica biosystems). The slides were

observed using a Zeiss Axioskop 40 and images were captured with an Axiocam IcC5 using Zeiss Axiovision software (version 4.8.2).

DNA quantification: DNA content was assessed before and after the decellularization process. DNeasy Blood & Tissue Kit (QIAGEN) was used according to the manufacturer's guidelines and as previously described [1, 2]. Briefly, the samples were digested overnight with Proteinase K at 56 °C followed by DNA purification and concentration was measured spectrophotometrically (Nanodrop, Thermo Scientific, US) by measuring the absorbance of the eluate at 260 nm.

Scanning Electron Microscopy (SEM).

Samples were fixed in 2.5% glutaraldehyde. Next, samples were washed 3 times with 1% PBS, placed in a 0.5% PBS solution containing 25% sucrose and 10% glycerol for 2 h and then snap frozen in liquid Nitrogen and process for SEM as previously described [1, 2]. Once frozen, samples were fractured, placed back into a 0.5% PBS solution containing 25% sucrose and 10% glycerol at RT for 1 hour. Once thawed, the samples were fixed with 1% Osmium tetroxide in 1% PBS at 3 °C for 1.5 hours, and washed with deionized water. Samples were then dehydrated through a series of dH_2O and ethanol baths until finally reaching 100% ethanol. Next, samples were dried using carbon dioxide and firmly fixed onto an aluminium stub with the fractured side opposite to the stub. This was followed by the addition of a 2 nm thick layer of Gold/ Palladium using a Gatan ion beam coater. Images were recorded with a 7401 FEG scanning electron microscope (Jeol, USA).

Second Harmonic Generation (SHG) and imaging.

Briefly, both native tissue and decellularized liver scaffolds were cryoprotected and sections were then set in a mould and covered in OCT cold embedding media and frozen as previously described [2]. The OCT blocks containing the liver tissue/scaffolds were cryosectioned to a thickness of 20 µm and mounted on glass slides. Prior to measurements, samples were thawed for 15 minutes in PBS. All SHG images were obtained using a custom built multiphoton microscope incorporating an upright confocal microscope (SP5, Leica) and a mode-locked Ti: Sapphire Laser (Mai Tai, Newport Spectra-Physics). Images of the SHG signal from collagen I were collected using an 820 nm excitation with SHG signal obtained with a 414/46 nm bandpass filter and multiphoton autofluorescence signal obtained with a 525/40 nm

bandpass filter. A 25X, 0.95 NA water-immersion objective (Leica) was used to deliver the excitation signal and to collect the SHG emission signal from the sample. Images with a 600 µm × 600 µm field of view were obtained with 2048 pixel resolution and a line rate of 10 Hz giving a pixel resolution of ~0.3 µm with 3X averaging on each acquisition to reduce the effect of noise.

Atomic Force Microscopy (AFM).

Sample preparation: Tissue samples for AFM measurement were prepared by taking tissue slices from a cube of liver tissue as previously described [1, 2]. Thick tissue slices were cut from the tissue cube using a scalpel, under liquid conditions and were kept in PBS at room temperature before attachment to a petri dish for analysis within 30 minutes. A slice of tissue measuring 5mm × 2 mm × 2 mm was attached to a petri dish using two droplets of Cyanoacrylate adhesive, applied with a 10 µl pipette tip, placed at the extremities of the sample. After tissue slice attachment (1–2 minutes) the slice was immersed in PBS in order for the AFM measurements to be conducted within a 2 hours time period. In the case of tissue scaffolds, samples were removed from storage in PBS at 4 °C and excess liquid removed with a tissue prior to attachment. As previously described for the tissue slices, tissue scaffold were attached to petri dishes with two droplets of adhesive at the extremities. After attachment (1–2 minutes) scaffolds were immersed in PBS and imaged within 2 hours.

AFM Measurements: Measurements of the tissue slices and scaffolds have been conducted on a JPK Nanowizard-1 (JPK Instruments) operating in force spectroscopy mode, mounted on an inverted optical microscope (IX-81, Olympus). AFM pyramidal cantilevers (MLCT, Bruker) with a spring constant of 0.07 N/m were used with a 35 µm glass bead attached to cantilever tip. Prior to measurements with the adapted cantilevers, their sensitivity was calculated by measuring the slope of the force-distance curve in the AFM software on an empty region of the petri dish. For indentation tests on the sample, the cantilever was aligned over regions in the middle of the samples using the optical microscope. For each sample 30–50 force curves were acquired in 6–10 different 100 µm regions, this arrangement allowed force-curves to be acquired in locations at least 50–100 µm apart. Force-curve acquisition was carried out with an approach speed of 5 µm/s and a maximum set force of 1.5 nN. Elastic moduli were calculated from the force-distance curves by fitting the contact region of the approach curve with the Hertz contact model, using the AFM software.

Proteomic analysis.

Protein extraction, separation and in-gel protein digestion: Proteins were extracted from healthy and cirrhotic liver tissues before and after the decellularization process, for every 3 biological repeats processed in triplicate [4]. Proteins were separated by 4-15% gradient SDS-PAGE, under reducing conditions and visualized by silver staining (ProteoSilver Plus, Sigma, UK). Bands (32 horizontal slices for each sample) were cut from the gel lane and de-stained using a solution containing 1:1 of 100 mM sodium thiosulphate and 30 mM potassium ferricyanide and incubated for 10 minutes at room temperature. Samples were reduced by 10 mM DTT and alkylated with 100 mM iodoacetamide using the ProGest Investigator Instrument (DigiLab, Genomics Solutions, Cambs, UK) according to the established protocol [5]. The dry gel pieces were rehydrated in 30 µL of 50 mM ammonium bicarbonate solution containing 0.2 ng/ µL of Trypsin Gold, Mass Spectrometry grade (Promega, Madison, USA) and incubated at 37°C overnight. After the tryptic digestion, any remaining solution, containing tryptic peptides and present in each tube, has been saved and each gel slice has been subjected to peptides extraction procedure. Briefly, each gel slice has been incubated, in sequence, with 3 different solutions having decreasing concentration of acetonitrile. At the end of each incubation, the solution was retained and pooled with the previous one and with the tryptic solution originally kept. In the end, pooled solutions for each gel band have been vacuum dried up to 20µl and the final volume adjusted with 0.1% formic acid, if needed.

Mass spectrometry: LC−MS/MS analysis was performed with an LTQ-Velos mass spectrometer (Thermo Fisher Scientific, UK). Peptide samples were loaded using a Nanoacquity UPLC (Waters, UK) with Symmetry C18 180 µm × 20 mm (Waters part number 186006527) trapping column for desalting and then introduced into the MS via a fused silica capillary column (100 µm i.d.; 360 µm o.d.; 15 cm length; 5 µm C18 particles, NikkyoTechnos CO, Tokyo, Japan) and a nanoelectrospray ion source at a flow rate at 0.42 µL/min. The mobile phase comprised H_2O with 0.1% FA (buffer A) and 100% acetonitrile with 0.1% FA (buffer B). The gradient ranged from 1% to 30% buffer B in 95 min followed by 30% to 60% B in 15 min and a step gradient to 80% B for 5 min with a flow of 0.42 µL/min. The full scan precursor MS spectra (400−1600 m/z) were acquired in the Velos-Orbitrap analyzer with a resolution of r = 60 000. This was followed by data dependent MS/MS fragmentation in centroid mode of the most intense ion from the survey scan using collision induced dissociation (CID) in the linear ion trap: normalized collision energy 35%; activation Q 0.25; electrospray voltage 1.4 kV; capillary

temperature 200°C; and isolation width 2.00. The targeted ions were dynamically excluded for 30 s, and this MS/MS scan event was repeated for the top 20 peaks in the MS survey scan. Singly charged ions were excluded from the MS/MS analysis, and XCalibur software version 2.0.7 (Thermo Fisher Scientific, UK) was used for data acquisition.

Protein identification: 96 Raw MS files (32 for each experimental replicate tested) per condition were loaded and assembled by MaxQuant (version 1.5.3) and visualized by Perseus (version 1.4.1.3) software platform. The peak list generated by Quant.exe (the first part of MaxQuant) was searched using the Andromeda search engine against rat FASTA files (RAT.fasta.gz) downloaded from the UNIPROT Website: ftp://ftp.uniprot.org/pub/databases/uniprot/current_release/knowledgebase/proteomes, last modified 19/2/2014. Selected parameters for Max-Quant analysis included the trypsin enzyme specificity, 2 missed tryptic cleavages, oxidation of methionine and acetylation of protein N-terminal as variable modifications and cysteine carbamidomethylation as fixed modification. In addition, the following parameters were applied to perform the database search: a minimum of 1 unique peptide identified for each protein, a peptide mass tolerance of 10 ppm for precursor ions, and a tolerance of 0.5 Da for MS/MS peaks. All proteins were filtered according to a false discovery rate (FDR) of 0.01% applied at both peptide and protein levels and a maximum peptide posterior error probability (PEP) of 1. MaxQuant output files were subsequently uploaded into Perseus in order to visualize and combine in one matrix the results obtained for the 3 different experimental conditions as well as to add GO terms for each protein group and to obtain data for the Venn Diagram. Moreover, the Label Free quantification algorithm, embedded into Max Quant, has been used to extrapolate relative protein quantification from analysed data.

Gene expression

EMT RT2 Profiler PCR Array: Total RNA was extracted using TRIzol reagent and RNeasy Universal Mini Kit (QIAGEN) according to the manufacturer's guidelines and previously described [2]. The quality and the quantity of the RNA was measured spectrophotometrically (Nanodrop, Thermo Scientific, US). RNA was pooled to a total yield of 1 µg from 3 biological replicates per sample condition and reverse transcription was performed using the RT^2 First Strand Kit (Qiagen). Complementary DNA templates for each sample were added to RT2 SYBR Green Mastermix and the resulting mixture was dispensed into the Epithelial to Mesenchymal

Transition RT² Profiler PCR Array (PAHS-090z, Qiagen, Hilden, Germany). The raw threshold cycle data for 3 cirrhotic and 3 healthy PCR arrays were uploaded onto the integrated web-based RT² Profiler PCR Array Data Analysis software package. All of the five housekeeping genes included in the EMT RT2 Profiler PCR Array, i.e. ACTB, B2M, GAPDH, HPRT1 and RPLPO had stable expression according to the stability scores calculated by DataAssist v3.0 software. The average (geometric mean) of these five endogenous control genes was used as the normalization factor to calculate the Delta Ct, using the formula Ct GOI – Ct HKG. Average fold changes in gene expression between the cells grown on cirrhotic 3D scaffolds and the healthy 3D scaffolds were calculated using ΔΔCt method in the PCR Array Data Analysis template using the following formula: 2^(-Avg(Delta(Ct)) of cirrhotic / 2^(-Avg(Delta(Ct)) of control (SABiosciences, Qiagen Company).

Protein synthesis and secretion.

Intracellular protein analysis: The repopulated snap-frozen scaffolds were washed in PBS1X and cells were lysed by placing scaffolds in radio-immunoprecipitation assay buffer (RIPA Buffer) [7] with 5mm diameter Stainless Steel Bead magnetic beads (Qiagen). The tubes were agitated for 5 minutes at full speed (50 cycles per second) using the TissueLyser (Qiagen). Total proteins were measured via micro-bicinchoninic (BCA) assay (Pierce, Rockford, IL, USA) according to the manufacturer's guidelines and stored at -80 °C for further analysis [7]. A 7-plex signalling assay for cell lysates was obtained by combining the TGF-β Signaling 6-plex Magnetic bead kit (48-614MAG), with a total β-tubulin Magnetic bead MAPmate (46-713MAG) for normalization. The analytes measured included SMAD2(Ser465/Ser467), SMAD3(Ser423/Ser425), ERK (Thr185/Tyr187), Akt (Ser473), total TGFβRII and total SMAD4. The assay was run as per manufacturer's instructions using 10 µg of total protein lysates. The signal was acquired by using the Luminex® MAGPIX® instrument and xPONENT® software was set to read the following bead regions: 35 (TGFBRII), 42 (ERK1/2), 43 (SMAD3), 47 (AKT), 64 (SMAD3), 67 (SMAD4), and 26 (tubulin). A minimum of 50 beads/region were counted and data were provided as mean fluorescence intensity (MFI), after blank subtraction. For each analyte and sample, data were normalized by dividing the net MFI for the corresponding tubulin net MFI.

TGFβ1 release in acellular scaffolds. Extracellular TGFβ1 levels in the culture media were measured with Human TGFβ1 ELISA Kit (ab100647) according to the manufacturer's

guidelines. Decellularized scaffolds of healthy and cirrhotic scaffolds, without cells, were incubated in culture media overnight. The absorbance of the samples was acquired on a FLUOstar® Omega Microplate Reader (BMG LABTECH) and analysed with MARS Data Analysis Software version 2.10.R3.

Fibronectin secretion: Secreted fibronectin levels were measured using an enzyme linked immunosorbent assay (ELISA), known as FBN-C, towards human fibronectin according to the manufacturer's guidelines (Nordic Bioscience A/S, Herlev, Denmark) [8]. Various time points were investigated i.e. day 0, 1 and 7 days and analysis was performed in duplicate for each sample (n=4 scaffolds for each condition).

Statistical analysis:

For the significance of differentially expressed protein the p-value Student's t-test was used and the sig-B [9]. Q-PCR results were expressed as mean ± s.d. data was analysed with ANOVA or Student's t-test. Two-tailed p values less than 0.05 were considered statistically significant. CPA data was considered non-parametric, distributions were described using median and quartiles and differences in populations tested with the Mann-Whitney U test.

Un-paired Student T test was used for the EMT profiler array dataset to compare the levels of each of the 84 genes of the EMT profiler between the healthy and the cirrhotic groups. Genes that presented expression levels with a fold change of 2 and $P \leq 0.05$ were considered to be differentially expressed.

References.

1. Mazza, G., et al., *Decellularized human liver as a natural 3D-scaffold for liver bioengineering and transplantation.* Sci Rep, 2015. **5**: p. 13079.
2. Mazza, G., et al., *Rapid production of human liver scaffolds for functional tissue engineering by high shear stress oscillation-decellularization.* Sci Rep, 2017. **7**(1): p. 5534.
3. Calvaruso, V., et al., *Computer-assisted image analysis of liver collagen: relationship to Ishak scoring and hepatic venous pressure gradient.* Hepatology, 2009. **49**(4): p. 1236-44.
4. Li, Q., et al., *Proteomic analysis of naturally-sourced biological scaffolds.* Biomaterials, 2016. **75**: p. 37-46.
5. Matyash, V., et al., *Lipid extraction by methyl-tert-butyl ether for high-throughput lipidomics.* J Lipid Res, 2008. **49**(5): p. 1137-46.
6. Schmittgen, T.D. and K.J. Livak, *Analyzing real-time PCR data by the comparative C(T) method.* Nat Protoc, 2008. **3**(6): p. 1101-8.
7. Longato, L., et al., *Reactive gamma-ketoaldehydes as novel activators of hepatic stellate cells in vitro.* Free Radic Biol Med, 2017. **102**: p. 162-173.

8. Bager, C.L., et al., *Quantification of fibronectin as a method to assess ex vivo extracellular matrix remodeling.* Biochem Biophys Res Commun, 2016. **478**(2): p. 586-91.
9. Cox, J. and M. Mann, *MaxQuant enables high peptide identification rates, individualized p.p.b.-range mass accuracies and proteome-wide protein quantification.* Nat Biotechnol, 2008. **26**(12): p. 1367-72.
