# Supplementary material for: Cirrhotic Human Liver Extracellular Matrix 3D Scaffolds Promote Smad-Dependent TGF-β1 Epithelial Mesenchymal Transition
Source: Cells. 2019 Dec 28;9(1):83. doi: 10.3390/cells9010083 (PMC7017194; doi:10.3390/cells9010083)
Supplement: Supplementary file 1 [file cells-09-00083-s001.zip › Supplementary Figure S1.pdf]

Figure S1

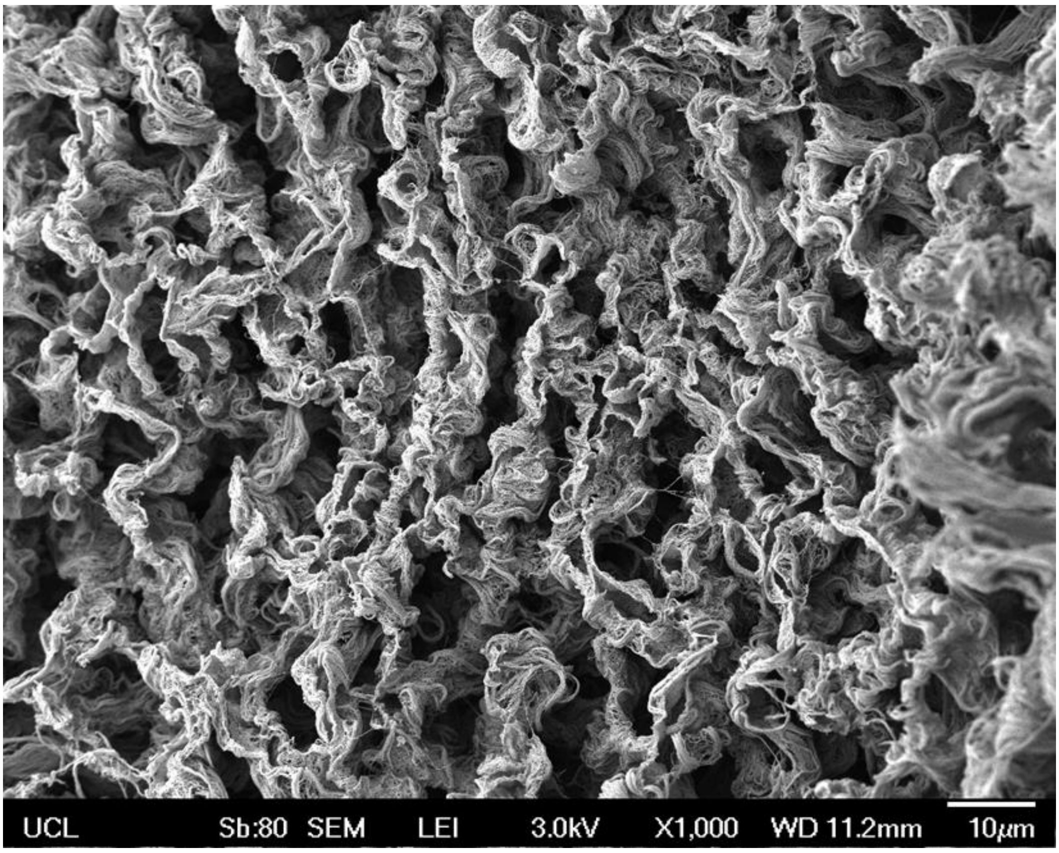

Figure S1. Microscopic characterization of decellularization of human healthy liver 3D scaffolds. Scanning Electron Microscopy of a decellularized 3D healthy scaffold showing the preservation of the thin collagen fibrils and hepatocyte pockets (octothorpe). Scale bars 10 µm.
