# Supplementary material for: Cirrhotic Human Liver Extracellular Matrix 3D Scaffolds Promote Smad-Dependent TGF-β1 Epithelial Mesenchymal Transition
Source: Cells. 2019 Dec 28;9(1):83. doi: 10.3390/cells9010083 (PMC7017194; doi:10.3390/cells9010083)
Supplement: Supplementary file 1 [file cells-09-00083-s001.zip › Giuseppe Mazza et al Table S1.pdf]

Table S1. Decellularization protocols followed for healthy and cirrhotic liver scaffolds.

Fresh reagents are used for each repetition. The Reagent Mix (RM) is composed of: 3% SDC, 0.5% SDS, 4.3% NaCl, 0.3% Triton X-100, 0.0025% EDTA and Milli-Q® ultrapure water (MQ).

Healthy scaffolds			Cirrhotic scaffolds		
Reagent	Duration (minutes)	Repetitions	Reagent	Duration (minutes)	Repetitions
MQ	2	5	MQ	2	20
NaCl	2	5	RM	2	1
MQ	2	5	RM	4	2
NaCl	2	5	PBS	1	1
MQ	2	3	NaCl	2	5
RM	2	1	MQ	2	5
RM	4	1	RM	2	1
PBS	5	3	RM	4	2
			MQ	2	10
			RM	2	1
			RM	4	2
			MQ	2	5
			RM	5	2
			MQ	2	5
			RM	5	2
			MQ	2	5
			RM	5	2
			PBS	5	3
