# Supplementary material for: Cirrhotic Human Liver Extracellular Matrix 3D Scaffolds Promote Smad-Dependent TGF-β1 Epithelial Mesenchymal Transition
Source: Cells. 2019 Dec 28;9(1):83. doi: 10.3390/cells9010083 (PMC7017194; doi:10.3390/cells9010083)
Supplement: Supplementary file 1 [file cells-09-00083-s001.zip › Giuseppe Mazza et al Table S2.pdf]

Table S2.

Antibodies used when performing immunohistochemistry and western blot analysis.

Antibody	Species	Dilution	Company	Catalogue N°
collagen I	Rabbit	1:200	Abcam	ab34710
collagen III	Rabbit	1:500	Abcam	ab7778
collagen IV	mouse	1:25	Dako	M0785
Laminin-α5-chain	mouse	1:200	Millipore	MAB1924
fibronectin	mouse	1:100	Millipore	MAB1937
TGFβ1	Rabbit	1:300	Abcam	ab92486
EPCAM	Mouse	1:50	Leica Biosystems	NCL-ESA
Alfa fetoprotein	Rabbit	1:200	Dako	A0008
Anti-mouse IgG-HRP	Goat	1:10000	Santa Cruz Biotechnology	Sc-2055
Anti-rabbit IgG-HRP	Goat	1:10000 or 1:5000	Cell Signalling Technology	#7074
